# Gastrointestinal basidiobolomycosis: a rare manifestation of *Basidiobolus ranarum* in a non-endemic region

**DOI:** 10.1093/jscr/rjae289

**Published:** 2024-05-03

**Authors:** Amany Fathaddin, Sarah Alobaid, Duaa Alhumoudi, Ghaida Almarshoud, Abdulaziz Alsubaie, Naif H Alotaibi

**Affiliations:** Department of Pathology, College of Medicine, King Saud University, Riyadh 12372, Saudi Arabia; College of Medicine, King Saud University, Riyadh 12372, Saudi Arabia; College of Medicine, King Saud University, Riyadh 12372, Saudi Arabia; College of Medicine, King Saud University, Riyadh 12372, Saudi Arabia; Department of Medicine, College of Medicine, King Saud University, Riyadh 12372, Saudi Arabia; Department of Medicine, College of Medicine, King Saud University, Riyadh 12372, Saudi Arabia

**Keywords:** gastrointestinal basidiobolomycosis, Basidiobolus ranarum, abdominal mass, voriconazole, Saudi Arabia

## Abstract

Gastrointestinal basidiobolomycosis (GIB) is a rare fungal infection caused by the *Basidiobolus ranarum*, and it possesses a significant challenge to diagnose it as it presents with non-specific symptoms that often mimic cancer. Herein, we report a case of GIB in a 51-year-old male from the central region of Saudi Arabia, a non-endemic region of GIB, which was initially misdiagnosed as colon cancer. A 51-year-old man presented with abdominal pain for two-months, non-bloody diarrhea, loss of appetite, and weight loss. Abdominal examination revealed a large mass measuring ~10x15cm. Radiological findings prompted the diagnosis of a colon mass, and the patient was surgically treated under that impression. Hemicolectomy and end colostomy with mucous fistula from distal sigmoid stump were done. Histopathology was consistent with GIB. The diagnosis of GIB presents a serious challenge and requires a high index of clinical suspicion.

## Introduction

Gastrointestinal basidiobolomycosis (GIB) is a rare and emerging fungal infection caused by the saprophyte *Basidiobolus ranarum* (*B. ranarum*) [1]. Worldwide reports of gastrointestinal infection by *B. ranarum* have been described amongst tropical and subtropical areas with some reports from Saudi Arabia, Iran, and Arizona in the United States of America [2]. In Saudi Arabia, the majority of reported cases were from the southern region, specifically Tohama [1, 2].

The disease typically manifests as a subcutaneous infection, and it is exceedingly rare for gastrointestinal tract to be affected [2]. In view of the rarity of the disease and lack of distinct clinical features, GIB is often misdiagnosed as it may mimic malignancy, irritable bowel disease (IBD), or tuberculosis [3].

Herein, we report a case of GIB in a 51-year-old male from the central region of Saudi Arabia, a non-endemic region of GIB, which was initially misdiagnosed and operated on as case of colon cancer.

## Case presentation

A 51-year-old gentleman from Al Qassim Province (Central Saudi Arabia) presented to the clinic complaining of left lower quadrant abdominal pain for two-months associated with non-bloody diarrhea, severe loss of appetite, and unintentional weight loss. Past medical history was remarkable for diabetes mellitus and hypertension. He had a family history of gastric/pancreatic cancer.

Abdominal examination revealed a firm mass in the left lower quadrant extending from the umbilicus medially to the anterior superior iliac spine laterally, and encompassing the entire area from top to bottom, including the groin. The mass was non-tender, immobile, and measuring ~ 10×15 cm.

**Figure 1:**
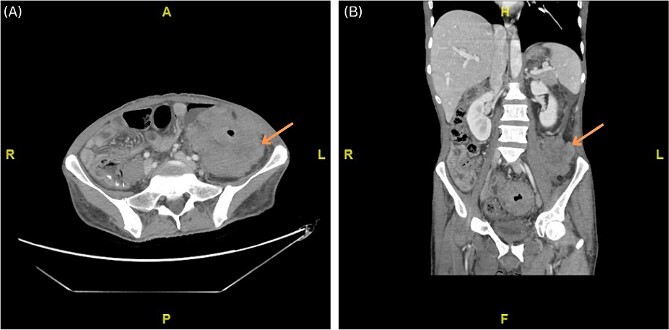
(A, B) Axial and coronal computed tomography (CT) scan showing marked non-uniform wall thickening and nodularity with heterogeneous enhancement invading the lateral part of the left psoas muscle and the left transverse abdominis muscle.

Abdominal CT scan with IV contrast displayed marked non-uniform wall thickening and nodularity with heterogeneous enhancement involving the mid rectum extending proximally to the splenic flexure of the colon, and multiple small size loco-regional lymphadenopathies (Fig. 1). Additionally, positron emission tomography (PET) scan displayed intense radiotracer uptake in the rectal, sigmoid and left colonic mass/wall. Further diagnostic evaluation included colonoscopy which revealed a rectosigmoid polyoid, friable, stricture-like, lesion, around ~15 cm in length (starting from 10 cm from anal verge and extending 25 cm upwards). Histopathology examination of the colonoscopy biopsies was inconclusive.

**Figure 2:**
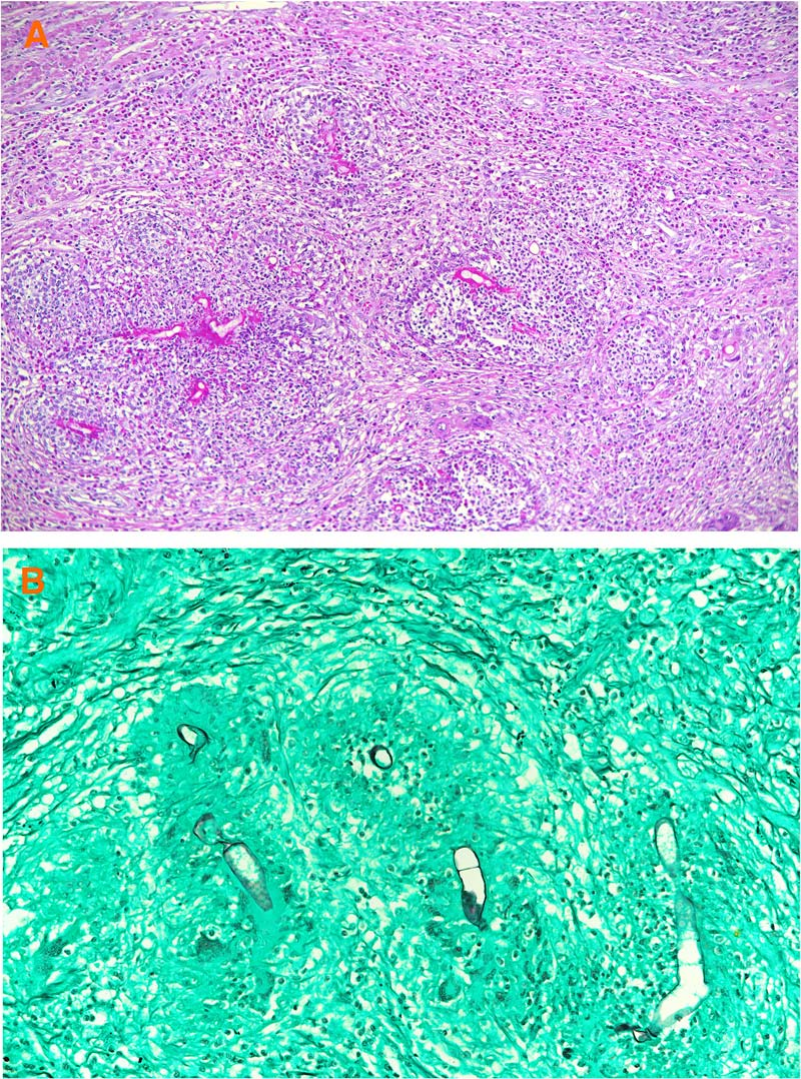
(A) Broad hyphae surrounded by eosinophilic material and a mixed inflammatory cell infiltrate containing multinucleated giant cells and numerous eosinophils. (A: H & E × 100), (B) Broad hyphae surrounded by eosinophilic material and a mixed inflammatory cell infiltrate containing multinucleated giant cells and numerous eosinophils. (B: GMS ×200).

Under the impression of a malignant mass, the patient underwent laparoscopic converted to open left hemicolectomy and end colostomy with mucous fistula from distal sigmoid stump. Microscopic examination of the hematoxylin and eosin (H & E) stained tissue sections from the resected left colon showed broad, septate hyphae surrounded by eosinophilic material and fungal zygospores in a background of marked mixed inflammation containing large number of eosinophils and multinucleated giant cells (Fig. 2A). Periodic acid-Schiff and gomori methenamine silver (GMS) special stains were performed and they intensified the fungal wall staining (Fig. 2B). The histopathologic morphology was consistent with colonic basidiobolomycosis. A tissue sample was sent to the national lab for polymerase chain reaction (PCR) confirmation which came back positive for *B. ranarum*.

After diagnostic evaluation and delving deeper into the patient’s history, it was discovered that he owns a bird sanctuary, and that he acquired a bird from Najran Province (Southern Saudi Arabia) shortly prior to developing the symptoms. The patient was started on voriconazole 400 mg IV twice a day, then was continued on oral voriconazole 200 mg twice a day for 6 months. Upon follow up in the clinic, the patient was doing well with an increase in appetite and weight. There was improvement in repeated radiological imaging, and his stoma is to be reversed soon.

## Discussion

Gastrointestinal basidiobolomycosis is a rare fungal infection that can affect both immunocompromised and immunocompetent hosts [4]. Internationally, GIB is mostly reported in tropical and subtropical areas [5]. Locally, in Saudi Arabia, there were few reported cases from the southern region [2]. In the present case, the patient lives in Al Qassim Province, in the central region of the kingdom, making it the first case to be reported in this region. Furthermore, our patient owns a bird sanctuary, and shortly prior to his presentation he acquired a bird from Najran Province (Southern Region). This raises a serious concern about the possibility of zoonotic transmission of the infection through birds.

Due to vagueness of presentation and lack of identifiable risk factors, GIB presents a diagnostic challenge and needs a high index of clinical suspicion [4]. GIB is often misdiagnosed as cancer, similarly to our case. The utility of radiological modalities in diagnosis of GIB is limited as it often displays non-specific findings such as masses and wall thickening resembling cancer or IBD [6]. *B. ranarum* is known to grow profoundly in submucosa or deep muscle layers, making endoscopic biopsies often inconclusive as well [6]. In our case, CT with contrast and endoscopic biopsies were both nonspecific for GIB and prompted the consideration of colonic cancer along with the PET scan results. Achieving a definitive diagnosis of GIB is done by culture on Sabouraud agar. In the majority of cases, however, GIB mimics cancer and patients eventually undergo surgical intervention under that impression, similarly to our patient [4]. Thus, intraoperative specimens are transferred to the lab in formalin, rendering isolation and culturing of the fungi unfeasible [4, 5]. Histopathologically, marked granulomatous inflammation, diffuse eosinophilic infiltrate, zygospores, and eosinophilic material surrounding the fungal element which is known is (Splendore–Hoeppli phenomenon) are the common features of *B. ranarum* [2, 4]. Despite PCR showing high sensitivity and specificity, it is not readily accessible due to the rarity of the infection [4].

Due to the rarity of the infection, devising the best treatment strategy is challenging. Combined surgical and medical treatment is the commonly used approach in the literature [2, 4]. However, relying solely on medical therapy was also reported to be successful in a few cases [7]. Serious complications of GIB have been reported. Death has been reported to occur in 18% of the cases [5].

To conclude, we presented a challenging case of GIB. The diagnosis of GIB presents a serious challenge and it requires a high index of clinical suspicion. While culture is the best diagnostic modality, histopathological diagnosis is more frequently employed. Long term management with azole antifungals is required to prevent relapse and further complications.

## Author contributions

Amany Fathaddin: Investigation – Histopathology, Writing – Review and Editing.

Sarah Alobaid: Writing – Original Draft, Writing – Review and Editing.

Duaa Alhumoudi: Writing – Original Draft, Writing – Review and Editing.

Ghaida Almarshoud: Writing – Original Draft, Writing – Review and Editing.

Abdulaziz Alsubaie: Conceptualization, Writing – Original Draft, Writing – Review and Editing.

Naif Alotaibi: Conceptualization, Supervision, Writing – Review and Editing.

## Conflict of interest statement

The authors declare no conflict of interest.

## Funding

The authors extend their appreciation to the Deanship of Scientific Research at King Saud University for funding this work through the Undergraduate Research Support Program.
